# Construction of a g-C_3_N_4_/Bi(OH)_3_ Heterojunction for the Enhancement of Visible Light Photocatalytic Antibacterial Activity

**DOI:** 10.3390/ijms25031872

**Published:** 2024-02-03

**Authors:** Jian Feng, Li Wang, Bo Xiao, Xia Ran, Caiying Wang, Jinming Zhu, Zuoji Liu, Chaozhong Li, Xinai Cui, Rong Li, Guangwei Feng, Zeqin Dai

**Affiliations:** 1 Engineering Research Center for Molecular Medicine, School of Basic Medical Sciences, Guizhou Medical University, Guiyang 550025, China; mona19851228@126.com (L.W.); xiaobogzmu@163.com (B.X.); ranxia@gmc.edu.cn (X.R.); caiyingwang2024@163.com (C.W.); jinmingzhu@gmc.edu.cn (J.Z.); liuzuoji@gmc.edu.cn (Z.L.); lichaozhong@gmc.edu.cn (C.L.); xinaicui@163.com (X.C.); lirong1@gmc.edu.cn (R.L.); fengguangwei@gmc.edu.cn (G.F.); 2 School of Pharmacy, Guizhou Medical University, Guiyang 550025, China

**Keywords:** g-C_3_N_4_/Bi(OH)_3_, *Escherichia coli*, photocatalytic antibacterial activity, visible light irradiation

## Abstract

Photocatalytic technology has been recently conducted to remove microbial contamination due to its unique features of nontoxic by-products, low cost, negligible microbial resistance and broad-spectrum elimination capacity. Herein, a novel two dimensional (2D) g-C_3_N_4_/Bi(OH)_3_ (CNB) heterojunction was fabricated byincorporating Bi(OH)_3_ (BOH) nanoparticles with g-C_3_N_4_ (CN) nanosheets. This CNB heterojunction exhibited high photocatalytic antibacterial efficiency (99.3%) against *Escherichia coli* (*E. coli*) under visible light irradiation, which was 4.3 and 3.4 times that of BOH (23.0%) and CN (28.0%), respectively. The increase in specific surface area, ultra-thin layered structure, construction of a heterojunction and enhancement of visible light absorption were conducive to facilitating the separation and transfer of photoinduced charge carriers. Live/dead cell staining, sodium dodecyl sulfate-polyacrylamide gel electrophoresis (SDS-PAGE) assays and scanning electron microscopy (SEM) have been implemented to investigate the damage to the cell membrane and the leakage of the intracellular protein in the photocatalytic antibacterial process. The e^−^, h^+^ and O_2_^•−^ were the active species involved in this process. This study proposed an appropriate photocatalyst for efficient treatment of bacterial contamination.

## 1. Introduction

At present, microbial contamination caused by bacteria or fungi has caught worldwide attention due to its serious threat to human health and aquatic ecosystems [1,2]. Antibiotic-resistant bacteria and fungi cause 2.8 million infections and 35,000 deaths each year in the United States. Various traditional technologies, including adsorption, filtration, flocculation, ozonation and chlorination, have been adopted to treat microbially contaminated water [2,3]. However, the toxic by-products, high cost, microbial resistance and long processing time of these traditional methods have limited their future practical large-scale application. Therefore, it is necessary to find an environmentally friendly, efficient and economical approach to eliminating pathogenic microorganisms from water. Photocatalytic technology can transmute solar energy into chemical energy via semiconductor photocatalysts [4,5]. It is regarded as one of the most promising strategies to resolve the environmental pollution problem faced by human society. So far, photocatalytic technology has also been employed to remove microbial contamination [6,7,8,9]. It has attracted widespread attention recently due to its unique features of nontoxic by-products, low cost, negligible microbial resistance and broad-spectrum elimination capacity [10,11,12]. In fact, the photocatalytic antibacterial process depends on the reaction between photogenerated reactive oxygen species (ROS) and bacteria [8]. And ROS originates from the reaction of photogenerated electron–hole pairs with water or dissolved oxygen in water. Therefore, the photocatalytic antibacterial performance of the catalyst is subject to its photocatalytic ability. Unfortunately, the photocatalytic ability of the catalyst is still constrained by slow migration, rapid recombination of photoinduced charge carriers and the low quantum efficiency of the photocatalytic reaction. The design and construction of a visible light-responsive photocatalyst with high quantum efficiency is the crucial challenge in realizing antibacterial applications.

Graphitized carbon nitride (g-C_3_N_4_, CN) is an emerging photocatalyst with a graphene-like layered structure. It possesses many remarkable advantages, like simple synthesis, superior biocompatibility, high chemical stability and low cost, which make CN an excellent candidate for antibacterial applications [13,14]. In addition, the composition, morphology, thickness and surface pore of CN can be readily regulated. It provides convenience for the synthesis of CN with different structures. Especially the moderate band position and proper band gap (~2.7 eV) make CN have excellent visible light response. Since its photocatalytic activity was reported in 2009, CN has become the focus of research in the area of photocatalysis [15]. However, the pristine CN still suffers from relatively low quantum efficiency, rapid recombination of photoinduced charge carriers and low surface-active sites [10]. So far, various CN-based photocatalysts have been constructed to overcome these drawbacks, such as heterojunction design [16,17], morphology control [18], doping [19], etc. The combination of CN with other compounds to form heterojunction has been regarded as the most efficient strategy to actualize visible light harvesting, ameliorate the separation efficiency of change carriers and ultimately enhance photocatalytic activity [20]. Considerable progress has been achieved recently in photocatalytic sterilization using CN-based heterojunction catalysts [21]. CeO_2_ [22], ZnFe_2_O_4_ [23], Co@ZnO [24], AgI [25], CuO [26], etc. have been adopted to form CN-based heterojunctions to acquire excellent photocatalytic antibacterial performance. For the practical application, however, there has still been a formidable challenge inboosting the photocatalytic antibacterial efficiency of CN-based heterojunction. As we previously reported [11], Bi(OH)_3_ is suitable for photocatalytic antibacterial applications. But its low visible light absorption and photocatalytic quantum efficiency cause a low yield of oxidizing radicals, which restricts the further practical antibacterial utilization of Bi(OH)_3_.

In this work, we first prepared porous CN nanosheets to increase theirspecific surface areas and surface-active sites. And then BOH nanoparticles were deposited on CN nanosheets. A CN/BOH 2D heterojunction photocatalyst was accordingly fabricated. The increase inspecific surface area, ultra-thin layered structure, construction of heterojunctions and enhancement of visible light absorption were conducive to facilitating the separation and transfer of photoinduced charge carriers. Then, the photocatalytic antibacterial performance of the CNB heterojunction was assessed by using Escherichia coli (*E. coli*) as the target bacteria. The destruction of the cell membrane, the leakage of the intracellular protein and the decomposition of the protein were considered events that occurred in the antibacterial process. This work supplied an optional scheme for the practical antibacterial application of the photocatalyst.

## 2. Results and Discussion

**Figure 1:**
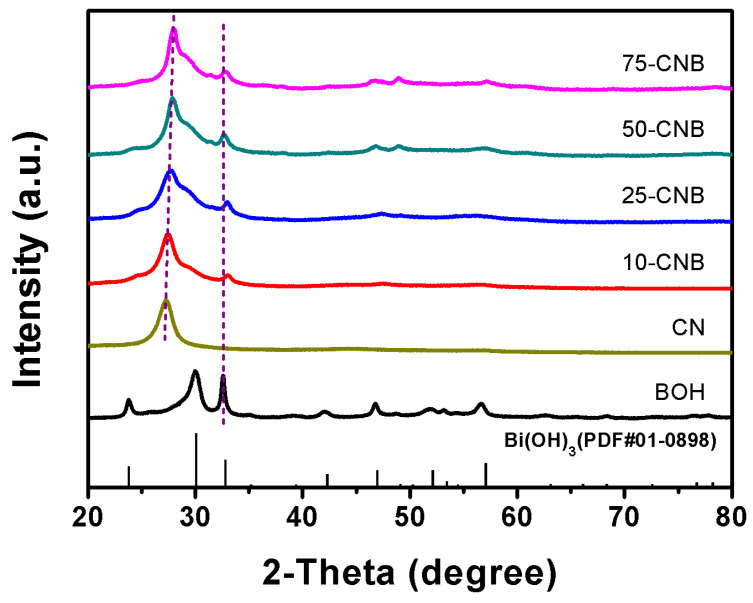
XRD patterns of CN, BOH and CNBs.

The X-ray diffraction (XRD) patterns of as-prepared CN, BOH and CNBs areshown in Figure 1. The XRD diffraction peaks of BOH at 23.90°, 30.17°, 32.90°, 42.40°, 47.05°, 52.23° and 57.17° were clearly observed, which could index to the pure Bi(OH)_3_ phase structure (PDF#01-0898) [27]. The characteristic diffraction peaks of g-C_3_N_4_ at 27.60° were visible in the XRD patterns of CN and CNBs, corresponding to the (002) crystal plane of g-C_3_N_4_. It indicated the presence of g-C_3_N_4_ in these samples. The diffraction peaks at 32.90° suggested that CNBs contained Bi(OH)_3_. Compared to pristine CN, the diffraction peak of CNBs at 27.60° gradually shifted to a larger angle as the BOH content increased. In addition, the peak of CNBs at 32.90° also shifted toward a large angleas compared with pure BOH. These changes revealed that BOH had been successfully incorporated into g-C_3_N_4_ [24]. This was important evidence for the formation of CNB heterojunctions. The average crystallite size of CN, 10-CNB, 25-CNB, 50-CNB, 75-CNB and BOH was estimated by using the Scherrer formula, which was 75, 77, 71, 103, 122 and 21.7 nm, respectively. The average crystallite size of CN and CNBs differed significantly from the particle size obtained using Transmission electron microscopy (TEM), as the Scheler formula is not applicable for the calculation of layered structures with a larger size than 100 nm.

**Figure 2:**
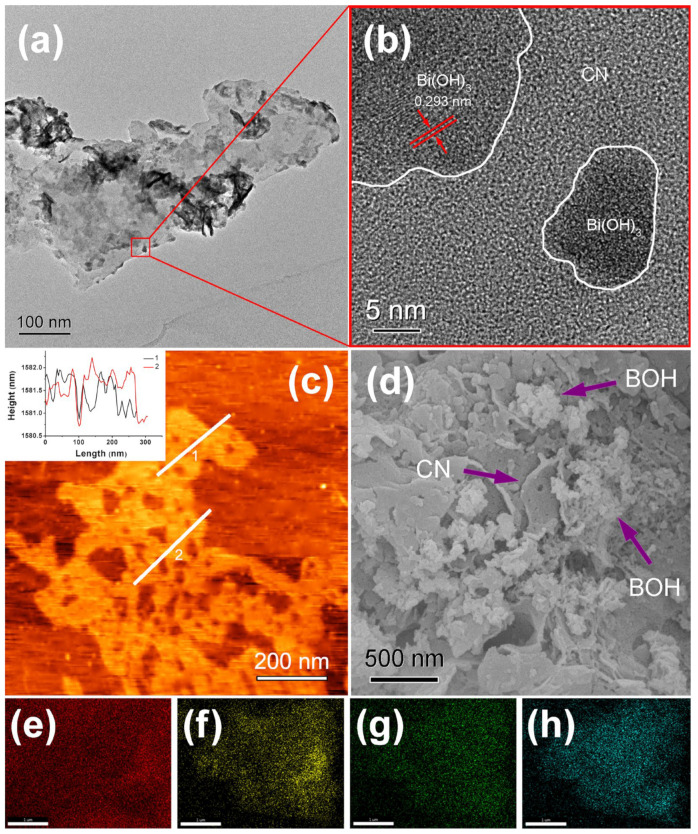
(**a**) TEM, (**b**) HRTEM, (**c**) AFM and (**d**) SEM images of 25-CNB, and corresponding element mappings of (**e**) C, (**f**) N, (**g**) O and (**h**) Bi. Scale bar = 1 mm (**e**–**h**).

The morphology of pristine CN could be observed using the TEM measurement (Figure S1). CN presented a large and thin-layered structure. The uneven pores were distributed on the surface of CN nanosheets. The pore diameter showed asignificant difference, which might be caused by the release of HCl and NH_3_ generated by the thermal decomposition reaction from NH_4_Cl during the synthesis process of CN [28]. The microstructure of 25-CNB was detected using TEM, high-resolution TEM (HRTEM), atomic force microscopy (AFM) and SEM. As shown in Figure 2a, irregular BOH particles were deposited unevenly on the surface of CN nanosheets. BOH particles exhibited a measure of aggregation. There was a great diversity in the particle size of BOH, which mainly resulted from the absence of a surfactant in the reaction solution. The lattice fringe space of 0.293 nm was achieved from the HRTEM image (Figure 2b), which well conformed to the corresponding crystal plane of Bi(OH)_3_. TEM and HRTEM results confirmed the compact contact between CN and BOH, which was a prerequisite for the formation of CNB heterojunctions.

The thickness of CN nanosheets was determined using AFM (Figure 2c), which was approximately 1.0 nm. The ultra-thin and porous layered structure of CN could reduce the diffusion distance of photoinduced charge carriers and increase its specific surface area and surface-active sites [22]. Therefore, it could ultimately improve its photocatalytic activity. The morphology of 25-CNB was also validated using SEM. As exhibited in Figure 2d, CN appeared to have a layered structure, and aggregated BOH particles were adhered to the CN surface. The elemental mapping results of 25-CNB demonstrated the existence of C, N, O and Bi in the as-prepared sample (Figure 2e–h). The element distribution of O and Bi indicated the uniform distribution of BOH on the CN surface.

**Figure 3:**
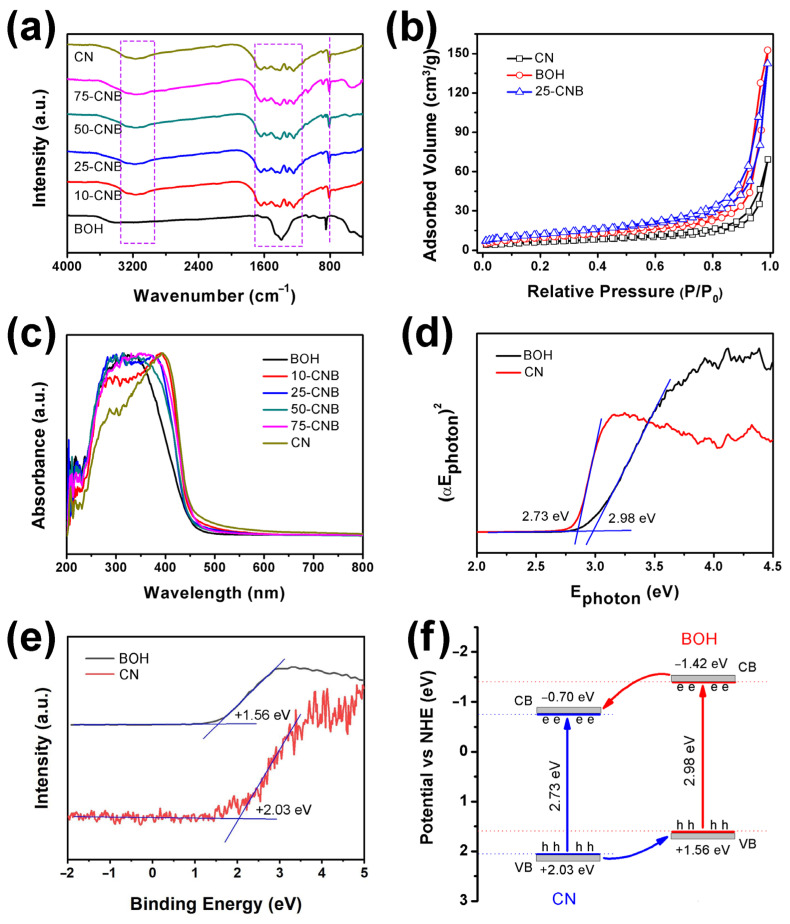
(**a**) FTIR transmittance spectra of CN, BOH and CNBs; (**b**) N_2_ adsorption–desorption isotherms of CN, BOH and 25-CNB; (**c**) DRS spectra of CN, BOH and CNBs; (**d**) band gap energies; (**e**) XPS VB spectra of CN and BOH and (**f**) a schematic illustration of the band gap structures of CNB.

Fourier-transform infrared spectra (FTIR) spectra were conducted to investigate the surface functional groups of the as-prepared CN, BOH and CNBs. As displayed in Figure 3a, the broad and weak peaks of CN and CNBs at 3000–3400 cm^−1^ (left dashed square in Figure 3a) were allocated to stretching vibrations of N-H from unpolymerized amino groups [29]. The characteristic peaks located between 1200 cm^−1^ and 1700 cm^−1^ (right dashed square in Figure 3a) were assigned to the skeletal vibrations in the tri-s-triazine heterocycle plane [30]. The peaks at 808 cm^−1^ (dashed verticle line in Figure 3a) were attributed to the breathing vibration of tri-s-triazine heterocycles and the deformation vibration of N-H [31]. The positions of these peaks were not obviously shifted after the formation of the CNB heterojunction, suggesting the CN structure was not damaged by the deposition of BOH. Additionally, the peak of pure BOH at 1390 cm^−1^ was allotted to the in-plane bending vibration of hydroxyl groups. The peak at 3430 cm^−1^ originated from the stretching vibration of hydroxyl. And the peak at 539 cm^−1^ might correspond to the stretching vibration of Bi-O bonds. The FTIR spectra of 50-CNB and 75-CNB showed weak peaks at 539 cm^−1^, implying the successful formation of the CNB heterojunction. However, these peaks could not be observed in 10-CNB and 25-CNB, which might result from the lower BOH content in these heterojunctions.

N_2_ adsorption–desorption isotherms of CN, BOH and 25-CNB were employed to assess its pore structures. The results are presented in Figure 3b. All isotherms exhibited a type IV feature, indicating these samples had mesoporous structures. The isotherm emerged in the H3 hysteresis loop when P/P_0_ was located in the range of 1–0.5, revealing the mesoporous structure of these samples was irregular [29]. The specific surface area, pore volume and average pore diameter obtained from N_2_ adsorption–desorption isotherms are listed in Table S1. The specific surface area and pore volume of CN were 22.82 m^2^ g^−1^ and 0.06 cm^3^ g^−1^, respectively. It was the smallest one among CN, BOH and 25-CNB. After BOH was deposited on the CN nanosheets, the specific surface area (43.59 m^2^ g^−1^) and pore volume (0.14 cm^3^ g^−1^) were significantly increased. The larger specific surface area and pore volume made 25-CNB possess more surface-active sites for facilitating adsorption of reactants and mass transfer during the photocatalytic process [22]. The BJH pore size distribution plots revealed a wide pore size range of CN, BOH and 25-CNB from 2 to 70 nm (Figure S2). This wide pore size distribution might arise from thermal aggregation during polymerization.

The optical properties of CN, BOH and CNBs were determined using the UV-Vis DRS spectra. As the results illustrated in Figure 3c show, CN and BOH had an absorption band edge of 454 and 416 nm, corresponding to band gaps (E_g_) of 2.73 and 2.98 eV, respectively. These E_g_ values were entirely consistent with the results calculated using the Kubelka–Munk function (Figure 3d). The E_g_ of CN coincided with the value reported in the literature [32]. In BOH, however, its E_g_ was much smaller than that of the reported Bi(OH)_3_ [27]. This might-originate from the differences in the structure of BOH due to our different synthesis conditions. These results indicated that CN had a higher visible light utilization efficiency than BOH. As shown in Figure 3c, all CNB heterojunctions exhibited higher visible light absorption in the wavelength range of 350–600 nm than pure BOH, indicating that CNBs might possess higher photocatalytic activity under visible light irradiation. The valance band (VB) positions of CN and BOH could be determined based on XPS VB spectra (Figure 3e). It was +2.03 and +1.56 eV for CN and BOH, respectively. Thus, the conduction band (CB) positions could be calculated by using the equation E_g_ = E_VB_ − E_CB_. It was found to be−0.70 and −1.42 eV, respectively. Therefore, the as-prepared CNBs belong to type II heterojunction (Figure 3f).

**Figure 4:**
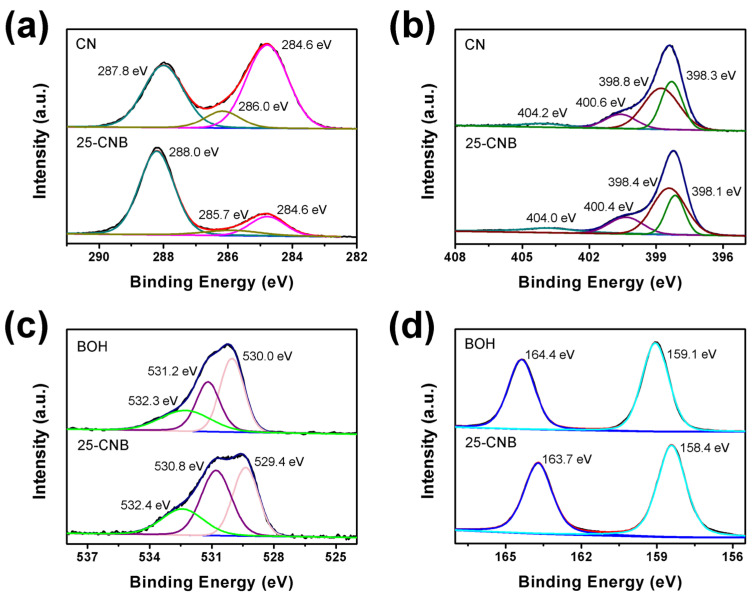
High-resolution XPS spectra of (**a**) C1s, (**b**) N1s, (**c**) O1s and (**d**) Bi4f.

The chemical composition, chemical valence and electronic environment of CN, BOH and 25-CNB were detected using the X-ray photoelectron spectroscopy (XPS) spectra. All the XPS spectra were calibrated by using the C1s peak (284.6 eV). The deconvoluted high-resolution XPS spectra of C1s, N1s, O1s and Bi4f were analyzed and are depicted in Figure 4. The peaks of C, N, O and Bi could be discriminated in survey spectra (Figure S3), corroborating the presence of these elements in as-prepared samples. The C1s peaks of CN were centered at 288.0, 286.2, and 284.6 eV (Figure 4a), which could be allocated to the C=N, C–N and C–C bonds of tri-s-triazine heterocycles, respectively [33]. The peaks at 288.0 and 286.2 eV were changed to 288.2 and 285.9 eV after depositing BOH on the CN surface. The N1s peaks of 25-CNB (Figure 4b) located at 404.0, 400.4, 398.4 and 398.1 eV could be assigned to π excitations C-NH_2_, N-(C)_3_ and C=N–C, respectively [23,28,34]. Interestingly, these peaks all shifted to low binding energy compared to CN. Commonly, the change in XPS peak position indicated the variation in the electronic environment on the surface of CN after the formation of heterojunctions. A similar variation in peak position could be observed in the XPS O1s and Bi4f spectra of BOH and 25-CNB as well [31]. These results demonstrated that a strong interaction between CN and BOH was generated in 25-CNB. This evidence directly confirmed the successful formation of the CNB heterojunction, which was consistent with the aforementioned XRD, TEM, SEM and element mapping results.

**Figure 5:**
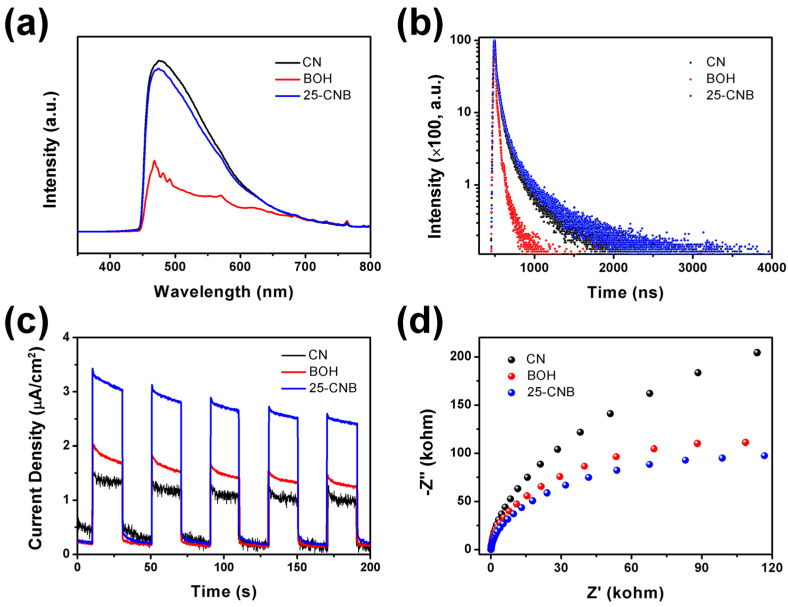
(**a**) PL spectra, (**b**) TRPL spectra, (**c**) TPC and (**d**) EIS spectra of CN, BOH and 25-CNB.

The separation and transfer properties of the photoinducted charge carriers could be investigated via photoluminescence spectra (PL), time-resolved photoluminescence spectra (TRPL), transient photocurrent response (TPC) and electrochemical impedance spectra (EIS) technologies. It is generally believed that the recombination of photoinduced charge carriers leads to fluorescence emission. The PL intensity can indirectly prove the recombination degree of photoinduced charge carriers inside the catalyst. The faster the recombination of charge carriers is, the higher the PL intensity. Thus, the concentration of charge carriers that can be used for photocatalytic reactions is relatively low, resulting in poor photocatalytic activity. Therefore, the PL intensity can be adopted to assess the separation capability of photoinduced charge carriers. As the PL spectra results displayed in Figure 5a show, pristine CN appeared to have the highest PL emission at 476 nm, implying that CN had the fastest recombination rate of charge carriers and the lowest photocatalytic activity. Once BOH was incorporated into CN, the PL intensity decreased obviously, indicating that the formation of the CNB heterojunction was beneficial for suppressing the recombination of the photoinduced charge carriers. BOH possessed the lowest PL intensity, which might result from the low excitation efficiency under light irradiation at 360 nm [22].

The TRPL spectra of CN, BOH and 25-CNB (Figure 5b) could be fitted using the biexponential decay equation. The details are listed in Table S2. The τ_1_ was of a short lifetime, which commonly representsthe radiation process. Comparatively, τ_2_ was of a long lifetime, standing for the non-radiation processes. TRPL results for25-CNB revealed that the percentage of short lifetime decreased while the percentage of long lifetime increased, compared to CN and BOH. It indicated that the formation of the CNB heterojunction effectively suppressed the radiative transition of charge carriers and facilitated theirnonradiative transitions. This was also the reason for the decrease in CNB PL intensity. Normally, the shorter the lifetime of the photoinducted charge carriers is, the faster the recombination rate becomes. The average PL lifetime of CN, BOH and 25-CNB was 5.92, 2.31 and 7.19 ns, respectively. 25-CNB exhibited the longest lifetime among these samples, suggesting that the relaxation time of charge carriers in 25-CNB was increased. These TRPL results confirmed that the recombination of the photoinduced charge carriers in 25-CNB was significantly inhibited.

As TPC results show in Figure 5c, 25-CNB manifested the highest photocurrent density among CN, BOH and 25-CNB under visible light irradiation, signifying that 25-CNB possessed the highest separation and transfer efficiency of charge carriers [35]. This was acutely beneficial for improving photocatalytic activity. Additionally, 25-CNB exhibited the smallest arc radius in the EIS Nyquist plots (Figure 5d), revealing that 25-CNB had the lowest resistance for the migration of photoinducted charge carriers [36]. This was more conducive to the migration of photoinducted charge carriers in 25-CNB to the surface of the catalyst and their participation in photocatalytic reactions. As such, 25-CNB might have the highest charge separation efficiency.

**Figure 6:**
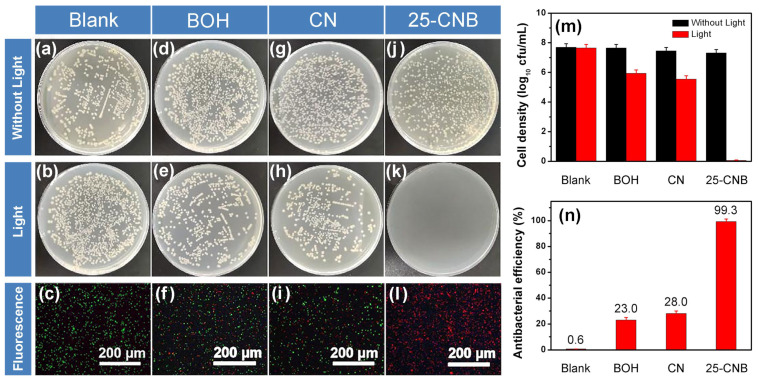
The photocatalytic antibacterial activity tested in the dark and under visible light irradiation and the corresponding live/dead cell staining of bacteria in (**a**–**c**) the blank, (**d**–**f**) BOH, (**g**–**i**) CN and (**j**–**l**) 25-CNB, respectively. (**m**) The bacterial colony counts in the dark and under visible light irradiation, respectively. (**n**) The photocatalytic antibacterial efficiency under visible light irradiation [Initial density: 7.7 × 10^7^ cfu/mL].

The photocatalytic antibacterial activity of the as-prepared CN, BOH and CNBs was evaluated by selecting *E. coli* as the target test microorganism under the irradiation of a white light LED. As shown in Figure S4a, for all samples, the number of bacterial colonies remained nearly constant without illumination (Figure 6), suggesting these samples exhibited excellent biocompatibility in the dark. The antibacterial efficiency of CN, BOH and CNBs increased with the extension of visible light irradiation (Figure S4b). After 2 h of irradiation, 25-CNB presented the highest antibacterial activity. Its bacterial clearance rate reached almost 100%. In the blank experiment, the number of bacterial colonies was not much impacted by lighting in the absence of a photocatalyst, revealing that visible light irradiation had a weak correlation with bacterial clearance. BOH and CN manifested certain photocatalytic antibacterial activity. As shown in Figure 6n, 23.0% and 28.0% of *E. coli* were inactivated after 2 h of irradiation over BOH and CN. As the live/dead cell staining results depicted in Figure 6f,i show, only a small portion of bacteria were killed under visible light irradiation. Importantly, the visible light-driven photocatalytic antibacterial efficiency of 25-CNB was dramatically increased (Figure 6k). No green fluorescence could be identified in Figure 6l, indicating the cell membrane of *E. coli* was almost completely destroyed after 2 h of visible light irradiation.

The recycling photocatalytic antibacterial experiments were conducted to assess the application perspective of CNBs. Five runs of antibacterial experiments with 25-CNB were implemented. As shown in Figure S5, the photocatalytic antibacterial activity of 25-CNB was almost invariable. Additionally, XRD patterns of 25-CNB before and after use were adopted to appraise its structural stability (Figure S6). No obvious change could be observed in the XRD result, indicating the stability of the crystalline structure of 25-CNB. Table S3 lists the photocatalytic antibacterial efficiencies against *E. coli* over different catalysts in recent research. It demonstrated that 25-CNB had outstanding visible light-driven photocatalytic antibacterial activity. These results indicated that CNBs possessed superior antibacterial activity, which mainly originated from their advantageous structure design. The ultra-thin and porous structure of CN nanosheets provided more surface-active sites and shorter migration distances for charge carriers. The construction of the CNB heterojunction promoted the separation and migration of charge carriers and suppressed their recombination. These structural characteristics made the improvement of the photocatalytic performance of CNBs possible.

**Figure 7:**
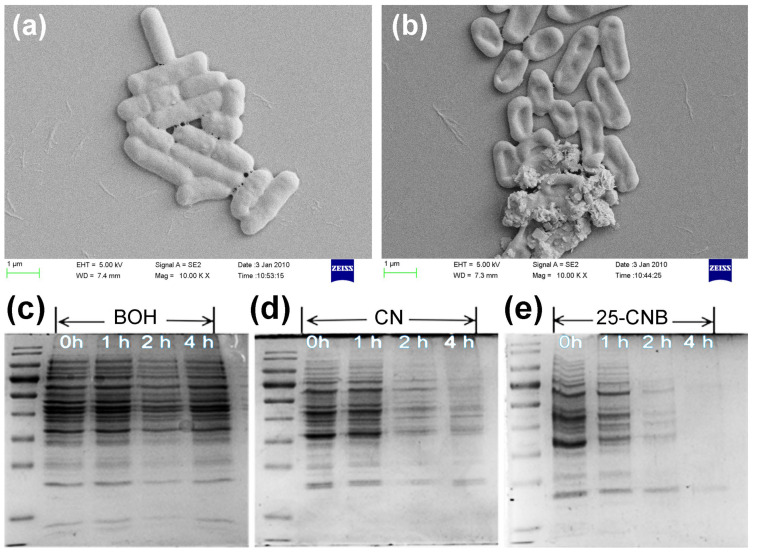
SEM images of *E. coli* (**a**) before and (**b**) after 2 h of visible light irradiation over 25-CNB. SDS-PAGE assay of the protein concentration treated with (**c**) BOH, (**d**) CN and (**e**) 25-CNB within different light irradiation times [initial density: 5 × 10^7^ cfu/mL].

Generally, the photocatalytic process can generate highly reactive superoxide and hydroxyl radicals, which will directly induce the destruction of the cell membrane, the leakage of the intracellular protein and the decomposition of the protein [11]. The cell membrane integrity of *E. coli* was confirmed by live/dead cell staining of the bacteria, as displayed in Figure 6c,f,i,l. Furthermore, the SEM image can also provide direct evidence to confirm the cell membrane damage. The SEM images of *E. coli* before and after 2 h of visible light irradiation over 25-CNB were illustrated in Figure 7. Before the photocatalytic treatment, the *E. coli* cells exhibited a smooth surface and rod-like shape (Figure 7a). It implied that *E. coli* cells were in a normal state and that their cell membrane was integral. After 2 h of irradiation, the cell membrane was seriously collapsed and evidently destroyed (Figure 7b). The destruction or perforation of the cell membrane could result in the leakage of the intracellular protein. The total protein released from *E. coli* was analyzed using SDS-PAGE measurement. As the results displayed in Figure 7c show, the intensity of the protein band remained nearly constant for BOH within 4 h of irradiation, signifying the protein concentration was not significantly decreased during the photocatalytic antibacterial process. By comparison, an evident descent of protein concentration was observed for CN and 25-CNB after 2 h of irradiation (Figure 7d,e), which indicated that CN and 25-CNB could cause the destruction of the *E. coli* cell membrane. This result also revealed that CN had slightly higher photocatalytic activity than BOH. The protein was hardly detectable after 4 h of visible light irradiation over 25-CNB (Figure 7e). It indicated that the leaked protein in the solution ultimately underwent photocatalytic decomposition. The intracellular protein concentration decreased from 470 μg mL^−1^ to 52 μg mL^−1^ after 4 h of visible light irradiation (Figure S7). This might result from the photocatalytic oxidation via the reactive oxygen species generated by the photocatalysis process.

**Figure 8:**
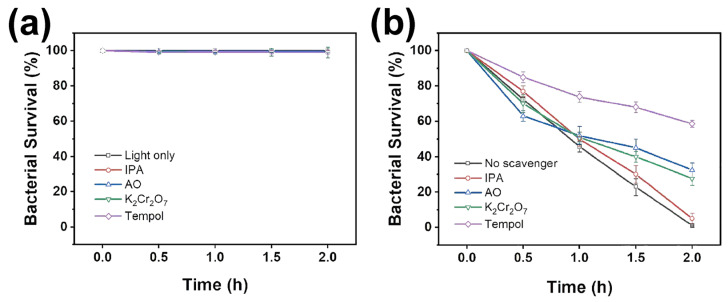
The effect of scavengers on the photocatalytic antibacterial efficiency under visible light irradiation (**a**) without and (**b**) with 25-CNB.

The active species mainly involved in the photocatalytic antibacterial process were analyzed by using K_2_Cr_2_O_7_, AO, TEMPOL and IPA as the scavengers to capture e^−^, h^+^, O_2_^•−^ and ^•^OH, respectively. The scavengers were first added alone to the *E. coli* solution to investigate their toxicity under visible light irradiation. As exhibited in Figure 8a, the bacterial survival rate was virtually invariable with 2 h of irradiation, suggesting these scavengers had low toxicity to *E. coli*. It also demonstrated that the reduction in bacterial survival rate in the presence of a photocatalyst was not due to the toxic effects of the scavengers. The antibacterial efficiency was tremendously reduced by adding TEMPOL to the *E. coli* solution (Figure 8b), implying that O_2_^•−^ radicals were the major active species that participated in the photocatalytic antibacterial process. In addition, the photocatalytic antibacterial efficiency was markedly decreased when K_2_Cr_2_O_7_ and AO were added. This manifested that e^−^ and h^+^ played minor roles in the photocatalytic antibacterial process. Comparatively, the presence of IPA just slightly influenced the antibacterial efficiency, suggesting that less ^•^OH was involved in the photocatalytic bactericidal process. Furthermore, DMPO was used as the trapping agent to capture the ^•^OH and O_2_^•−^ in the photocatalytic process to form DMPO-^•^OH and DMPO-O_2_^•−^. The ESR signals of DMPO-^•^OH and DMPO-O_2_^•−^ were recorded to assess the production of the radicals under visible light irradiation. As exhibited in Figure S8, no ESR signals were observed in the dark. After 15 min of visible light irradiation, DMPO-^•^OH and DMPO-O_2_^•−^ signal intensities were notably enhanced, indicating the significant production of hydroxyl radicals and superoxide radicals under illumination.

Based on the results discussed above, a photocatalytic antibacterial process could be proposed. The CNB heterojunctions were constructed via the deposition of BOH particles on the surface of CN nanosheets (testified by the XRD, TEM, HRTEM and XPS results). The specific surface area and absorption capacity for visible light were improved after the formation of the CNB heterojunction (certified by the BET and UV-Vis DRS spectra). The as-prepared CNBs belonged to type II heterojunction (evidenced via UV-Vis DRS and XPS VB spectra). The photoinduced electrons and holes were produced under visible light irradiation. The photoinduced electrons in the CB of BOH were transferred to the CB of CN. At the same time, the photoinduced holes in the VB of CN migrated to the VB of BOH (Figure 3f). The CB potential of CN was negative enough, which can reduce O_2_ to form O_2_^•−^ [37]. Furthermore, the VB potential of BOH was lower than that of the redox potential of ^•^OH/OH^−^ (1.99 eV vs. NHE) [38], indicating that no hydroxyl radical could be generated on BOH. It only might be produced on CN, which resulted in the concentration of OH^−^ in the *E. coli* solution being very low. That might be why adding IPA had barely an impact on antibacterial efficiency in the radical capture experiment. Consequently, e^−^, h^+^ and O_2_^•−^ were the active species in the photocatalytic antibacterial process. PL, TRPL, TPC and EIS spectra confirmed that CNB heterojunctions possessed higher separation efficiency than photoinduced charge carriers. Actually, the increase in specific surface area, the ultra-thin layered structure, the construction of the heterojunction and the enhancement of visible light absorption should be the reasons why the CNB heterojunction has high photocatalytic antibacterial activity.

## 3. Material and Methods

### 3.1. Sample Preparation

NH_4_Cl, dicyandiamine, ethylene glycol, Bi(NO_3_)_3_, NaOH and absolute ethanol were purchased from Aladdin Chemical Technology Co. Ltd. (Shanghai, China). All chemicals are analytical grade and used without any further purification.

Preparation of porous CN nanosheets: Firstly, 5 g NH_4_Cl and 5 g dicyandiamine were mixed and calcined at 550 °C for 4 h. Bulk CN was obtained after cooling it down to room temperature. The bulk CN was then ground in an agate mortar. A total of 100 mg of ground CN was mixed with 200 mL of deionized water. After 4 h of sonication, the supernatant was separated by centrifugation and evaporated by lyophilization to acquire porous CN nanosheets.

Preparation of the CNB heterojunction: For the synthesis of the CNB containing 10 wt% Bi(OH)_3_ (named 10-CNB), 100 mg of CN nanosheets were dissolved using ultrasound in 40 mL of a mixture solution composed of a 1:1 ratio of ethylene glycol (EG) and deionized water. Then 18.7 mg of Bi(NO_3_)_3_·5H_2_O was added. The pH of the reaction solution was adjusted to 11 using a 2 M NaOH solution. After reacting for 4 h, the product was collected by vacuum filtration and washed with absolute ethanol and deionized water several times. Subsequently, the product was dried at 60 °C in a vacuum for 10 h. 25-CNB, 50-CNB, 75-CNB and BOH were similarly synthesized by adjusting the dosage of Bi(NO_3_)_3_·5H_2_O and CN.

### 3.2. Characterization

X-ray diffraction (XRD) patterns were tested using the Rigaku Smartlab diffractometer (Rigaku Corporation, Tokyo, Japan). Transmission electron microscopy (TEM) and high-resolution TEM (HRTEM) images were recorded using the JEOL-2100F transmission electron microscope (JEOL, Tokyo, Japan). Scanning electron microscopy (SEM), energy dispersion spectrum (EDS) and elemental mapping images were measured using the JSM-4800F scanning electron microscope (JEOL, Tokyo, Japan). Atomic force microscopy (AFM, Bruker, Billerica, MA, USA) images were taken using the Bruker Multimode 8 AFM system (NanoScope Analysis v180r1). Fourier-transform infrared spectra (FTIR) were obtained using the Nicolet NEXUS 470 spectrometer (ThermoFisher, Waltham Mass, MA, USA) at room temperature through the KBr pellet method at 400–4000 cm^−1^. X-ray photoelectron spectroscopy (XPS) was measured using the Thermo ESCALAB 250XI system (ThermoFisher, Waltham, MA, USA). The N_2_ adsorption desorption isotherms and BET-specific surface area were analyzed using the ASAP 2460 Micrometrics instrument (Micrometrics, Londonderry, NH, USA). The ultraviolet–visible diffuse reflectance spectra (DRS) were determined using the Shimadzu UV-2401 spectrophotometer (Shimadzu Corporation, Aichi, Japan).

### 3.3. Photocatalytic Antibacterial Activity

The photocatalytic antibacterial activity of the as-prepared sample was evaluated by selecting *E. coli* as the target test microorganism. All relevant containers and culture medium were sterilized at 121 °C for 20 min. *E. coli* were incubated in a liquid medium at 37 °C for 12 h. The supernatant was removed by centrifugation and washed three times using PBS with pH = 7.0. The bacterial concentration was adjusted to 5 × 10^7^ cfu/mL by the gradient dilution of PBS. A total of 4 mg of photocatalyst was dispersed in 20 mL of bacterial suspension. Then the suspension was illuminated with a 40 W LED for 20 min. The bacterial suspension was diluted using the decimal method. A total of 100 μL of bacterial suspension was evenly coated on the LB solid medium and incubated at 37 °C for 18–24 h. The number of colonies on the culture dish after each sterilization experiment was counted. Each experiment was conducted three times, in parallel.

### 3.4. Live/Dead Cell Staining of Bacteria

The bacteria treated with the catalyst for different irradiation times (0, 0.5, 1.0, 1.5, 2.0 h) were resuspended with 0.9% NaCl. A certain amount of bacterial suspension was fetched out and stained with N01/PI (Live/Dead Bacteria Staining Kit, BB-41266, Bestbio, Shanghai, China). The fluorescent images were taken using fluorescent microscopy.

### 3.5. Analysis of Total Protein

The leakage of total protein from the bacteria in the process of photocatalytic sterilization was analyzed to assess the antibacterial activity of the as-prepared sample. After a certain period of visible light treatment, 40 mL of bacterial solution (ca. 5 × 10^7^ cfu/mL) was centrifuged, and the supernatant was discarded. A total of 500 μL of PBS was added for resuspension. This bacterial solution was sonicated with a cell crusher for 30 min and then centrifuged at 4 °C for 15 min. The supernatant was taken to obtain the membrane protein. According to the instructions of the BCA Elisa kit (P0010S, Beyotime Biotechnology Co., Shanghai, China), 20 μL of membrane protein and 200 μL of BCA working solution were added into 96-well plates and incubated at 37 °C for 20 min. The absorbance at 562 nm was recorded using a microplate reader, and the protein concentration was calculated according to the protein standard curve. SDS-PAGE technology was used to further observe the dynamic change in the concentration of *E. coli* protein. A 160 μL protein sample and 40 μL 5 × Protein loading buffer were mixed and boiled for 5 min. A 20 µL protein sample was loaded into the wells of the gel. A 12% SDS-PAGE was conducted in Tris Glycine SDS buffer at 120 V. The protein bands were stained with Coomassie R-250 (B105005, Aladdin, Shanghai, China) overnight, washed with water and then added to a decolorant solution to disperse and decolor until the protein bands were clear.

### 3.6. Radical Capture Experiment

The scavengers were used to explore the active species in the photocatalytic bactericidal process under the irradiation of a 40 W LED for 20 min. Isopropanol (IPA), 4-hydroxy-2,2,2,6,6-tetramer hydroxypiperidine epoxy (TEMPOL), ammonium oxalate (AO) and potassium dichromate (K_2_Cr_2_O_7_) were selected as the scavengers to trap ^•^OH, O_2_^•−^, h^+^ and e^−^, respectively. The concentration of each scavenger was kept appropriate to ensure that it was nontoxic to *E. coli* [39].

### 3.7. Photoelectrochemical Measurement

The photoelectrochemical experiments with the as-prepared catalyst were implemented on the CHI 660E electrochemical workstation. Pt and Ag/AgCl were utilized as the counter electrode and reference electrode. For the preparation of the working electrode, 5 mg of the as-prepared catalyst was mixed first with 0.2 mL of Nafion in 1.8 mL of ethanol by sonication and then dropped on 1 cm^2^ of FTO glass. The electrochemical impedance spectroscopy (EIS) of the catalyst was detected in a 0.2 M Na_2_SO_4_ electrolyte solution.

## 4. Conclusions

In summary, a novel 2D g-C_3_N_4_/Bi(OH)_3_ heterojunction was successfully constructed using a facile method at room temperature. The as-prepared CNBs presented an improvement in their photocatalytic antibacterial activity. The antibacterial efficiency against *E. coli* under visible light irradiation was up to 99.3%, which achieved an immense improvement over that of BOH (23.0%) and CN (28.0%). The increase in specific surface area, the ultra-thin layered structure, the construction of the heterojunction and the enhancement of visible light absorption were conducive to facilitating the separation and transfer of photoinduced charge carriers. It should be the reason that CNB had a high photocatalytic antibacterial activity. Additionally, e^−^, h^+^ and O_2_^•−^ were the active species involved in the destruction of the cell membrane, the leakage of the intracellular protein and the decomposition of the protein. This work elaborates on the relationship between the CNB heterojunction structure and its photocatalytic antibacterial performance, which provides an alternative photocatalyst for the treatment of practical bacterial contamination.

## Data Availability

The original data are available from the corresponding author upon reasonable request.

## Supplementary Materials

The supporting information can be downloaded at: https://www.mdpi.com/article/10.3390/ijms25031872/s1. References [40,41,42,43,44,45,46,47] are cited in the Supplementary Materials.
