# Supplementary material for: Construction of a g-C3N4/Bi(OH)3 Heterojunction for the Enhancement of Visible Light Photocatalytic Antibacterial Activity
Source: Int J Mol Sci. 2024 Feb 3;25(3):1872. doi: 10.3390/ijms25031872 (PMC10856445; doi:10.3390/ijms25031872)
Supplement: Supplementary file 1 [file ijms-25-01872-s001.zip › ijms-2794795-supplementary.pdf]

Construction of g-C_3N_4/$Bi(OH)_3$ heterojunction for the enhancement of visible light photocatalytic antibacterial activity

Jian Feng*, Li Wang, Bo Xiao, Xia Ran, Caiying Wang, Jinming Zhu, Zuoji Liu, Chaozhong Li, Xinai Cui, Rong Li, Guangwei Feng and Zeqin Dai*

Engineering Research Center for Molecular Medicine, School of Basic Medical Science/School of pharmacy, Guizhou Medical University, Guiyang 550025, China

* Corresponding author. Tel.: +86 851 88174017
E-mail address: jfeng@gmc.edu.cn (Jian Feng); daizeqin300@163.com (Zeqin Dai)

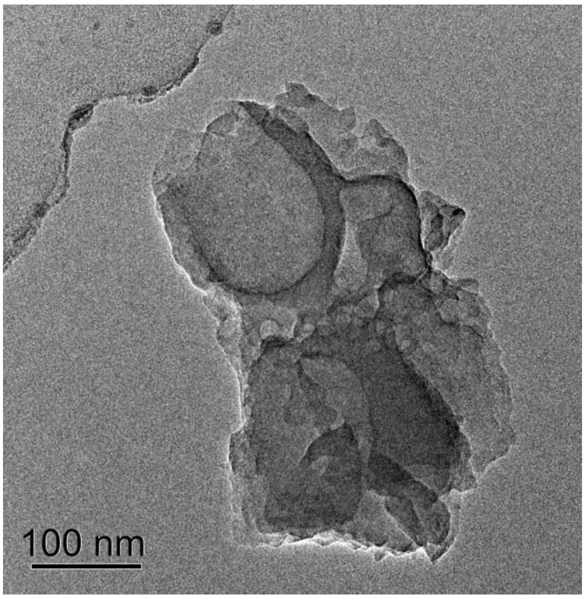

Fig. S1 TEM image of pristine CN

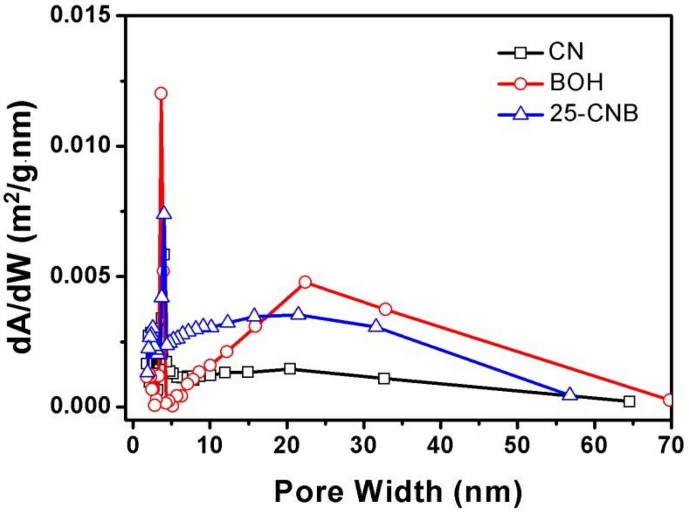

Fig. S2 The BJH pore size distribution plots of CN, BOH and 25-CNB

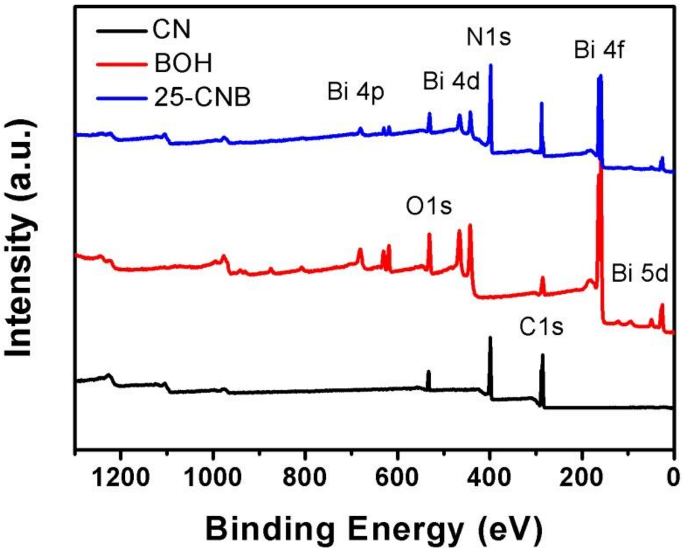

Fig. S3 XPS survey spectra of CN, BOH and 25-CNB

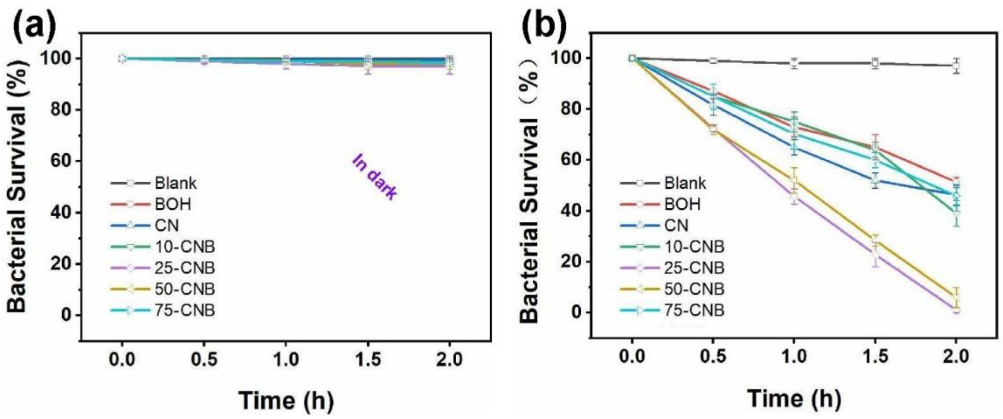

Fig. S4 The photocatalytic antibacterial efficiency of CN, BOH and CNB heterojunctions a) in dark and b) under visible light irradiation

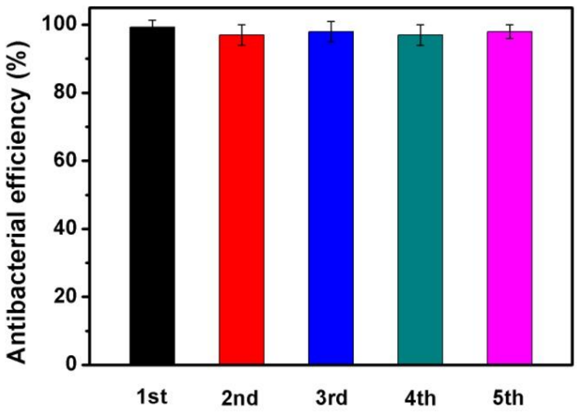

Fig. S5 The recycling photocatalytic antibacterial experiments against *E. coli* with 25-CNB

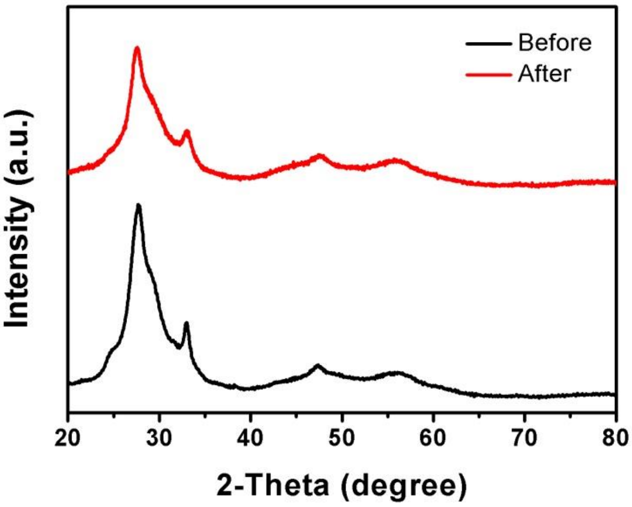

Fig. S6 XRD patterns of 25-CNB before and after recycling experiments

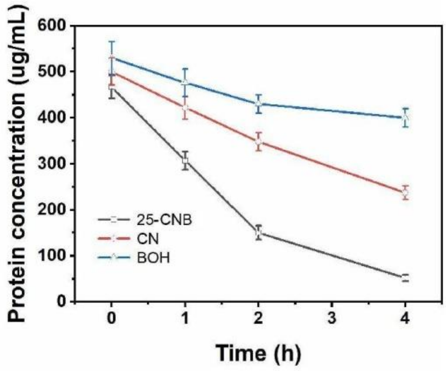

Fig. S7 The protein concentration treated by CN, BOH and 25-CNB within different light irradiation time

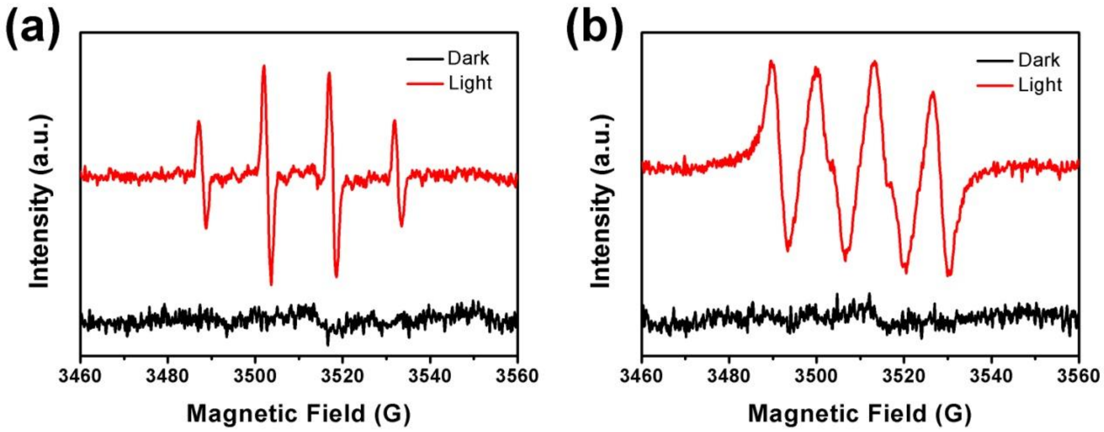

Fig. S8 The ESR spectra of a) DMPO-•OH and b) DMPO-•O_2^- in photocatalytic process over 25-CNB in dark and under 15 min of visible light irradiation

Table S1. The summarized results obtained from N_2 adsorption-desorption isotherms.

Sample	V_{pore} (cm^3/g)	D_{pore} (nm)	S_{BET} (m^2/g)
CN	0.06	17.26	22.82
BOH	0.16	22.79	34.72
25-CNB	0.14	18.68	43.59

Table S2. The fitted parameters obtained from the decay curves of TRPL

Sample	τ_1 (ns) (Rel. %)	τ_2 (ns) (Rel. %)	τ_a (ns)
CN	3.64 (87.6)	22.0 (12.4)	5.92
BOH	1.73 (94.9)	13.0 (5.1)	2.31
25-CNB	4.23 (85.7)	24.9 (14.3)	7.19

Table S3 Comparison of photocatalytic antibacterial activity of 25-CNB with various photocatalysts

Photocatalysts	Light source	Antibacterial efficiency %	Reference
g-C_3N_4/$Bi(OH)_3$	White LED (40 W)	99.3 (2 h, *E. coli*)	this work
$BiSnSbO_6$-TiO_2	LED lamp (35 W)	71.33 (3 h, *E. coli*)	[1]
P,S-g-C_3N_4	Xenon lamp (300 W)	97.0 (2.5 h, *E. coli*)	[2]
Fe_3O_4-TiO_2	Solar light	87.2 (2 h, *E. coli*)	[3]
GaN:ZnO	Xenon lamp (300 W)	94 (2 h, *E. coli*)	[4]
Ag/Ag_2S/rGO	Xenon lamp (300 W)	97.76 (24 h, *E. coli*)	[5]
Bi/C_3N_4	Xenon lamp (300 W)	96.4 (1 h, *E. coli*)	[6]
$Ni(PO_3)_2$/C	Halide lamp (70 W)	71.9 (1 h, *E. coli*)	[7]
GQDs/NH_2-MIL-125	LED lamp (100 W)	92 (1 h, *E. coli*)	[8]

References

[1] Chen, J.; Shan, M.; Shi, X.; Zhang, S.; Li, J.; Luan, J.; Duan, L.; Hou, H. $BiSnSbO_6$–TiO_2 composites enhance LED light-driven photocatalytic antibacterial activity. *Ceram. Int.* **2022**, *48*, 19036-19046.

[2] Fang, Y.; Pei, S.; Zhuo, L.; Cheng, P.; Yuan, H.; Zhang, L. Phosphorus and sulfur codoped carbon nitride nanosheets with enhanced photocatalytic antibacterial activity and promotion of wound healing. *Appl. Surf. Sci.* **2022**, *586*, 152761.

[3] Ma, S.; Zhan, S.; Jia, Y.; Zhou, Q. Superior Antibacterial Activity of Fe_3O_4-TiO_2 Nanosheets under Solar Light. *ACS Appl. Mater. Interfaces* **2015**, *7*, 21875–21883.

[4] Leng, B.; Zhang, X.; Chen, S.; Li, J.; Sun, Z.; Ma, Z.; Yang, W.; Zhang, B.; Yang, K.; Guo, S. Highly efficient visible-light photocatalytic degradation and antibacterial activity by GaN:ZnO solid solution nanoparticles. *J. Mater. Sci. Technol.* **2021**, *94*, 67–76.

[5] Huo, P.; Liu, C.; Wu, D.; Guan, J.; Li, J.; Wang, H.; Tang, Q.; Li, X.; Yan, Y.; Yuan, S. Fabricated Ag/Ag_2S/reduced graphene oxide composite photocatalysts for enhancing visible light photocatalytic and antibacterial activity. *J. Ind. Eng. Chem.* **2018**, *57*, 125–133.

[6] Zhang, H.; Li, L.; Li, Q.-Q.; Ma, T.; Gao, J.-Q.; Xue, J.-B.; Gao, S. Graphitic carbon nitride loaded with bismuth nanoparticles displays antibacterial photocatalytic activity. *Rare Met.* **2022**, *41*, 1570–1582.

[7] Zhang, X.; Sun, G.; Jia, S.; Xie, H.; Kang, Z.; Chen, W.; Cui, M.; Wang, B.; Wang, B.; Chen, X.; Yang, D.-P. Intrinsic carbon defects induced nickel phosphate/carbon photocatalyst for high performance bacterial disinfection. *Chem. Eng. J.* **2022**, *438*, 135624.

[8] Yang, G.; Wang, L.; Zhang, C.; Li, P.; Du, H.; Mao, Y.; Qiu, M.; Li, Q.; Hao, D.; Wang, Q. Novel graphene quantum dots modified NH_2-MIL-125 photocatalytic composites for effective antibacterial property and mechanism insight. *Sep. Purif. Technol.* **2023**, *312*, 123433.
